# Dysuricemia

**DOI:** 10.3390/biomedicines11123169

**Published:** 2023-11-28

**Authors:** Akiyoshi Nakayama, Masafumi Kurajoh, Yu Toyoda, Tappei Takada, Kimiyoshi Ichida, Hirotaka Matsuo

**Affiliations:** 1 Department of Integrative Physiology and Bio-Nano Medicine, National Defense Medical College, Tokorozawa 359-8513, Japan; 2 Department of Metabolism, Endocrinology and Molecular Medicine, Graduate School of Medicine, Osaka Metropolitan University, Osaka 545-8585, Japan; 3 Department of Pharmacy, The University of Tokyo Hospital, Tokyo 113-8655, Japan; 4 Department of Pathophysiology, Tokyo University of Pharmacy and Life Science, Hachioji 192-0392, Japan

**Keywords:** monosodium urate (MSU), crystal formation, pro-oxidative effects, anti-oxidative effects, neurodegenerative diseases (NDs), Parkinson’s disease (PD), Alzheimer’s disease (AD), chronic kidney disease (CKD), cardiovascular disease (CVD), bucket-and-balls theory

## Abstract

Gout results from elevated serum urate (SU) levels, or hyperuricemia, and is a globally widespread and increasingly burdensome disease. Recent studies have illuminated the pathophysiology of gout/hyperuricemia and its epidemiology, diagnosis, treatment, and complications. The genetic involvement of urate transporters and enzymes is also proven. URAT1, a molecular therapeutic target for gout/hyperuricemia, was initially derived from research into hereditary renal hypouricemia (RHUC). RHUC is often accompanied by complications such as exercise-induced acute kidney injury, which indicates the key physiological role of uric acid. Several studies have also revealed its physiological role as both an anti-oxidant and a pro-oxidant, acting as both a scavenger and a generator of reactive oxygen species (ROSs). These discoveries have prompted research interest in SU and xanthine oxidoreductase (XOR), an enzyme that produces both urate and ROSs, as status or progression biomarkers of chronic kidney disease and cardiovascular disease. The notion of “the lower, the better” is therefore incorrect; a better understanding of uric acid handling and metabolism/transport comes from an awareness that excessively high and low levels both cause problems. We summarize here the current body of evidence, demonstrate that uric acid is much more than a metabolic waste product, and finally propose the novel disease concept of “dysuricemia” on the path toward “normouricemia”, or optimal SU level, to take advantage of the dual roles of uric acid. Our proposal should help to interpret the spectrum from hypouricemia to hyperuricemia/gout as a single disease category.

## 1. Introduction

The novel disease concept of “dysuricemia” was proposed for the first time as our symposium title in 2019 [1] to help interpret the spectrum from hypouricemia to hyperuricemia/gout as a single disease category.

Uric acid, which is ionized as urate in serum, tends to be regarded as simply a metabolic waste product since uric acid is the end metabolite of purine bodies in humans [2,3]. It has a pathogenic effect on gout flare, caused by the formation of monosodium urate (MSU) crystals after prolonged hyperuricemia, that is, an elevated serum urate (SU) level [4,5]. The conventional wisdom tends to be “the lower, the better”, similar to advice on low-density lipoprotein (LDL) cholesterol levels [6].

**Figure 1:**
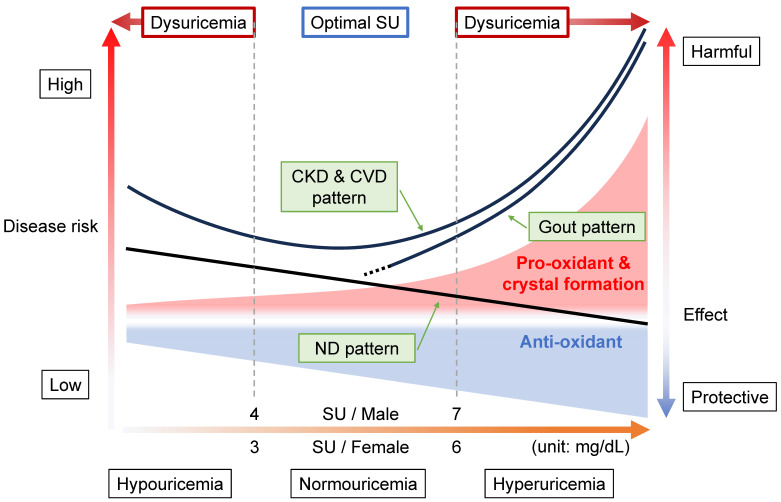
Disease concept of dysuricemia. The horizontal axis indicates serum urate (SU) level. The vertical axes on the left side and right side, respectively, indicate the disease risks and harmful/protective effect sizes of uric acid (ionized as urate in serum). Because uric acid has a dual role (providing an anti-oxidative protective effect and a pro-oxidative and/or crystal-forming harmful effect), both of these effect sizes increase as a function of rising SU level. The harmful effects increase more steeply with increasing SU level than the increase in protective effect due to the formation of monosodium urate crystals, which are deposited due to the low solubility (6.8 mg/dL, or approximately 400 μmol/L) of urate in human serum. There are three typical patterns of disease risk associated with SU level. In the “gout pattern”, increasing SU results in a higher disease risk of gout due to crystal formation, while neurodegenerative diseases (NDs) such as Parkinson’s disease show the “ND pattern”, indicating a lowered disease risk with rising SU level due to its neuroprotective effects. The risks for chronic kidney disease (CKD) and cardiovascular disease (CVD) generate a J-curve due to the combination of gout and ND patterns, the “CKD & CVD pattern”. As shown here, “dysuricemia” includes both hyperuricemia and hypouricemia. To reduce the burdens of several common diseases, the dual nature of uric acid should be exploited by maintaining normouricemia, with the optimal SU level being 4 < SU ≤ 7 mg/dL (240 < SU ≤ 420 μmol/L) for males and 3 < SU ≤ 6 mg/dL (180 < SU ≤ 360 μmol/L) for females.

However, uric acid is also known to have a dual role in oxidative stress in humans: both an anti-oxidative protective effect and a pro-oxidative and/or a harmful crystal-forming effect [7]. Indeed, a hereditary disease, renal hypouricemia (RHUC), is sometimes accompanied by exercise-induced acute kidney injury (EIAKI), which is estimated to be caused by not-scavenged oxidative stress [8]. Figure 1 shows why the concept of “dysuricemia”, which consists of hyperuricemia and hypouricemia, is necessary.

As described below, considerable research on the relationship between SU level and several common disease risks has revealed characteristic patterns. We broadly classify them here into three patterns in connection with the dual effects of uric acid. In the first pattern, the “gout pattern”, increased SU causes a higher disease risk caused by the formation of monosodium urate crystals due to the low solubility (6.8 mg/dL; approximately 400 μmol/L) of uric acid in human serum [9]. With the second pattern, the “neurodegenerative disease (ND) pattern”, the disease risk is lower, as a high SU level increases the neuroprotective effect of uric acid. Neurodegenerative diseases, including Alzheimer’s disease (AD) and Parkinson’s disease (PD), are common disorders that can result in dementia, and their risk patterns are similar to those seen with SU. Thirdly, there is the “chronic kidney disease (CKD) and cardiovascular disease (CVD) pattern”, in which disease risks follow J-curves in parallel with the SU level. This is likely because both CKD and CVD are vascular-related diseases and are affected by the dual effect of uric acid, that is, of the above-described gout and ND patterns.

As shown in Figure 1 and described below, “dysuricemia”, which includes both hyperuricemia and hypouricemia, influences several diseases in humans. In short, “the lower, the better” is incorrect; the ideal is to maintain normouricemia, or optimal SU level, to reduce the burdens of several common diseases with dysuricemia.

In this review, the dual role of uric acid is described with its benefits and drawbacks to support our rationale for having created this novel disease category of “dysuricemia”.

## 2. History of Dysuricemia

Gout is one of the oldest disease concepts. First identified by the Egyptians in 2640 BC, “podagra”, or gout, was later recognized by Hippocrates in the fifth century BC [10]. He reported that the risk factors of gout are being male, advanced age, obesity, and alcohol consumption [11]. Six centuries later, Galen added heredity as a gout risk [10]. In 1679, Antonie van Leeuwenhoek used his microscope to observe needle-shaped crystals taken from a gouty tophus [10,12], of which the components were later revealed by Wollaston in 1797 to be uric acid [13]. Garrod showed elevated SU concentration in gout patients in 1848 [14], and hyperuricemia has since been established as the cause of gout. Although primary gout was in the past seen mostly in a few wealthy people, it is increasingly a globally common and challenging disease [15]. Population-based studies from Asia, Europe, and North America have reported its incidence to range between 0.6 and 2.9 per 1000 person-years and a prevalence of 0.68–3.90% in adults [4,5]. Indigenous Asian and Pacific people such as Taiwanese and Māori are reported to have a much higher prevalence, partly and possibly due to the protective effect of uric acid against malaria [16]. The Japanese population is reported to have experienced genetic selection pressure for gout susceptibility over the last 2000–3000 years [17].

The first case study of a patient showing hypouricemia [8] was reported by Praetorius et al. in 1950 [18]. Although there was a misunderstanding about the effect of pyrazinamide in a report by Greene et al. [19], they reported a case study on the characteristics of hereditary disorders of urate reabsorption at the renal tubules in 1972; three years later, Akaoka et al. [20] first reported it in Japan. Ishikawa et al. [21] named the concomitant kidney injury with RHUC as “ALPE” (Acute renal failure with severe Loin pain and Patchy renal ischemia after anaerobic Exercise) from its symptoms and have since researched it in detail. More recently, ALPE is also being called “EIAKI”. Although it is a rare hereditary disease worldwide, it is relatively frequent in Japanese, Korean, and Eastern European Roma populations [8,22,23,24] and is also observed in Chinese and Jewish populations [22,25]. Approximately 0.3% of the Japanese population is estimated to have hypouricemia caused by RHUC [8,26]. The few causal variants of RHUC that are widely shared in Japanese patients can be explained by the “founder effect”, i.e., most of the present Japanese population is descended from a very small ancestral population with RHUC variants [27].

It is of interest that research on patients with RHUC has provided new insights into uric acid handling and has resulted in the identification of therapeutic molecular targets for gout and hyperuricemia [28].

## 3. Production of Uric Acid and Dysuricemia

**Figure 2:**
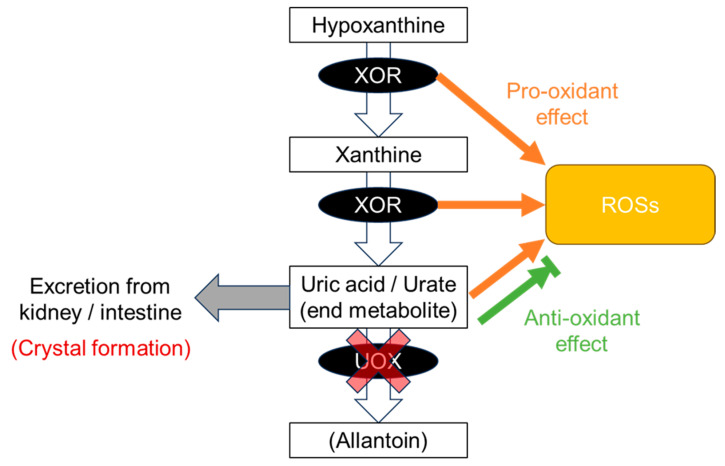
Production of uric acid. At the end of the purine metabolic pathway, uric acid is produced from hypoxanthine and xanthine by xanthine oxidoreductase (XOR). Uric acid is an end product in humans due to a defect in urate oxidase (UOX) and is excreted from the kidney and intestine. Along this pathway, XOR produces reactive oxygen species (ROSs) as a pro-oxidative effect. Uric acid has both anti-oxidative and pro-oxidative effects against ROS. Slightly soluble uric acid, which has low solubility in human serum, occasionally forms crystals before being excreted from the body.

In humans, xanthine oxidoreductase (XOR) is chiefly expressed in the liver and intestine [29] and is the rate-limiting enzyme in uric acid production [30], catalyzing the oxidation of hypoxanthine to xanthine and xanthine to uric acid in the purine metabolism pathway (Figure 2). Although urate is then catalyzed by uricase, also known as urate oxidase (UOX), to allantoin in most mammals, uric acid is the end product of purine metabolism in humans due to two dysfunctional variants in the *UOX* gene [2,3] and is considered a cause of hyperuricemia and gout in humans.

The XOR protein is a homodimer consisting of a subunit composed of three domains linked by hinge regions: two unequal iron–sulfur clusters (2Fe/S) in the N-terminal domain, a flavin adenine dinucleotide (FAD) cofactor in the intermediate domain, and a molybdopterin cofactor containing a molybdenum atom (Moco) in the C-terminal domain.

XOR activity in the liver has been reported to be higher in gouty patients with overproduction of uric acid [31], and XOR is a therapeutic target for the treatment of patients with hyperuricemia and gout caused by overproduction of uric acid [32]. Recent studies have shown that plasma XOR activity is higher in males, obese individuals, and those with insulin resistance and is also positively associated with SU levels, suggesting that increased XOR activity may contribute to the hyperuricemia often seen in these individuals via increased production of uric acid [33,34,35].

Xanthinuria, which was first described by Dent and Philpot in 1954 [36], is another asymptomatic primary hypouricemia caused by a genetic defect in xanthine dehydrogenase (*XDH*/*XOR*) [37] for xanthinuria type 1 (XAN1; Mendelian Inheritance in Man (MIM) 278300) [38], or of the molybdenum cofactor sulfurase (*MOCOS*) for xanthinuria type 2 (XAN2; MIM 603592) [39]. Xanthinuria is characterized by marked hypouricemia and reduced urinary excretion of uric acid in addition to increased urinary excretion of xanthine as a result of reduced XOR activity, but its frequency is very low, with somewhat more than 150 cases reported worldwide. Xanthine has low solubility and can form xanthine stones, but patients have no other serious symptoms [37], possibly due to the decreased pro-oxidative and anti-oxidative effects of XOR and urate.

XOR is an enzyme that is involved not only in the production of uric acid but also in ROS. XOR is a constitutive NAD^+^-dependent xanthine dehydrogenase (XDH) that can be reversibly converted to xanthine oxidase (XO) by oxidation of two cysteine residues or irreversibly by partial proteolysis of the fragment containing these cysteine groups [40]. The XO form produces superoxide ion (O2^•−^) and hydrogen peroxide (H_2_O_2_) by monovalent and divalent electron transfer to O_2_, respectively, whereas the XDH form produces these ROSs at the FAD site by acting as a nicotinamide adenine dinucleotide hydrate (NADH) oxidase [7]. Plasma XOR activity has been reported to affect blood pressure [41], glycemic control [42], renal function [43], coronary artery spasm [44], vascular endothelial function [45], and carotid atherosclerosis [46], suggesting that ROS production by XOR is involved in these pathologies.

Urate has shown both anti-oxidative and pro-oxidative properties in vitro by scavenging and the production of ROSs [47,48], and both hypouricemia and hyperuricemia, or dysuricemia, appear to contribute to CKD and CVD due to an imbalance between pro-oxidant, crystal formation, and anti-oxidant characteristics as described below. In addition to these effects of urate, the impact of XOR-mediated ROS production requires further investigation.

## 4. Urate Transporters and Dysuricemia

**Figure 3:**
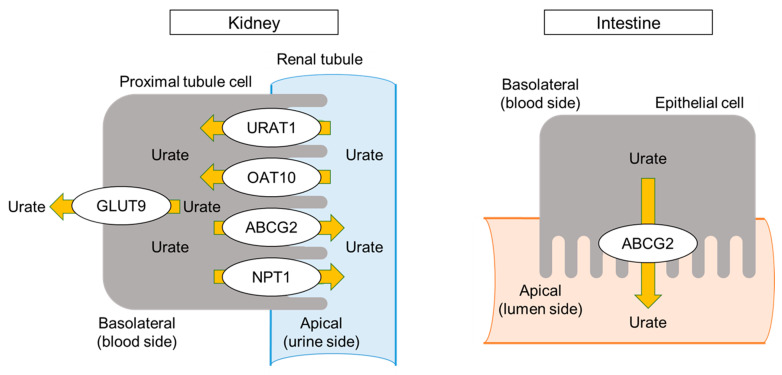
Urate transporters. Serum urate (SU) level is regulated by maintaining a balance between uric acid production and urate transport (excretion and reabsorption) in the kidney and intestine. The urate transporters shown here were identified by both genetic and functional studies: *URAT1*/*SLC22A12*, *GLUT9*/*SLC2A9,* and *OAT10*/*SLC22A13* genes encode transporters for urate reabsorption, while *ABCG2*/*BCRP* and *NPT1*/*SLC17A1* encode urate excretion transporters on the renal proximal tubular cells. ABCG2 is also expressed in the intestinal epithelial cells as the main urate exporter.

Urate, the end metabolite, is then transported to and excreted from the kidney and intestine. This results in stronger pathophysiological effects by transporters than by metabolic effects on dysuricemia when compared to those of dyslipidemia and diabetes mellitus. Figure 3 shows urate transporters encoded by *URAT1*/*SLC22A12*, *GLUT9*/*SLC2A9*, *ABCG2*/*BCRP*, *NPT1*/*SLC17A1,* and *OAT10*/*SLC22A13*, all of which have been shown by both genetic and functional studies to have pathophysiological roles in dysuricemia [49,50,51,52,53]. Of these, URAT1, GLUT9, and ABCG2 are characteristic transporters whose dysfunctional variants strongly affect SU levels.

Urate transporters URAT1 and GLUT9 have been identified in RHUC patients [49,50]. RHUC is caused by dysfunction of urate reabsorption in the kidney, and RHUC patients show low SU (typically ≤ 2 mg/dL or 120 μmol/L) and high fractional excretion of uric acid (FE_UA_) [8]. RHUC itself is asymptomatic, as is hyperuricemia, but its complications, such as ALPE, also known as exercise-induced acute kidney injury (EIAKI), and urolithiasis, are sometimes evident. Patients’ median onset age of EIAKI is reported to be 19 years old, and the male-to-female ratio is 10:1 [28]. From the sequence similarity to *OAT4*/*SLC22A11*, the *URAT1*/*SLC22A12* gene was identified. It encodes the urate reabsorption transporter at the apical side of the renal proximal tubule cells, and its dysfunctional variants, such as rs121907892 (p.W258X), cause RHUC type 1 (RHUC1; MIM 220150) [49]. The *GLUT9*/*SLC2A9* gene encodes urate reabsorption at the basolateral side of the renal proximal tubule cells, and its dysfunction causes RHUC type 2 (RHUC2; MIM 612076) [50]. Both RHUC1 and RHUC2 in patients with two nonfunctional variants cause severe to moderate hypouricemia (SU ≤ 2.0 mg/dL or 120 μmol/L); however, RHUC2 cases have higher FE_UA_ (typically >100%) than that of RHUC1 patients (typically 25–90%) [54]. Selective URAT1 inhibitors such as dotinurad and lesinurad have recently been developed as uricosuric agents to treat gout and hyperuricemia.

The common dysfunctional variants of the *ABCG2*/*BCRP* gene, rs72552713 (p.Q126X) and rs2231142 (p.Q141K), have been identified as the main genetic cause of the common form of the disease (that is, primary gout/hyperuricemia) [51,55] as well as early-onset gout [56]. ABCG2 is a high-capacity urate exporter [51] and is expressed on the apical side of the renal proximal tubule cells, enterocytes, and hepatocytes in humans [57]. Because dysfunctional ABCG2 more strongly decreases extra-renal (intestinal) urate excretion than renal excretion, ABCG2 dysfunction increases renal excretion overall, and it appears as an overproduction of uric acid [58]. Based on this novel pathogenesis of hyperuricemia, Ichida et al. [58] propose that the “overproduction type” be renamed “renal overload type,” consisting of two subtypes, “extra-renal urate underexcretion” and genuine “overproduction” of uric acid (Table 1). Decreased intestinal excretion has been proven not only in *Abcg2* knockout mice [58] but also in humans; indeed, both gastroenteritis and hemodialysis patients showed decreased intestinal and renal urate excretion, respectively, in parallel to their ABCG2 dysfunction levels [59]. In other words, dysfunctional *ABCG2* variants are the shared cause of hyperuricemia due to both renal overload and underexcretion of urate [60]. NPT1/SLC17A1 is also a urate exporter that is located in the renal proximal tubules in humans, and its common gain-of-function missense variant, rs1165196 (p.I269T), causes gout with renal underexcretion [52] by increasing urate transport without changing its expression levels [61]. A dysfunctional missense variant rs117371763 (p.R377C) of *OAT10/SLC22A13,* which encodes urate absorber, has also been shown to decrease both gout risk and SU levels [53] by increasing FE_UA_ [62]. Renal OAT10 inhibition might be involved in the urate-lowering effect of losartan and lesinurad, which exhibit uricosuric effects; losartan inhibits OAT10 more strongly than URAT1 [62].

## 5. “Bucket-and-Balls” Theory for Hyperuricemia

**Figure 4:**
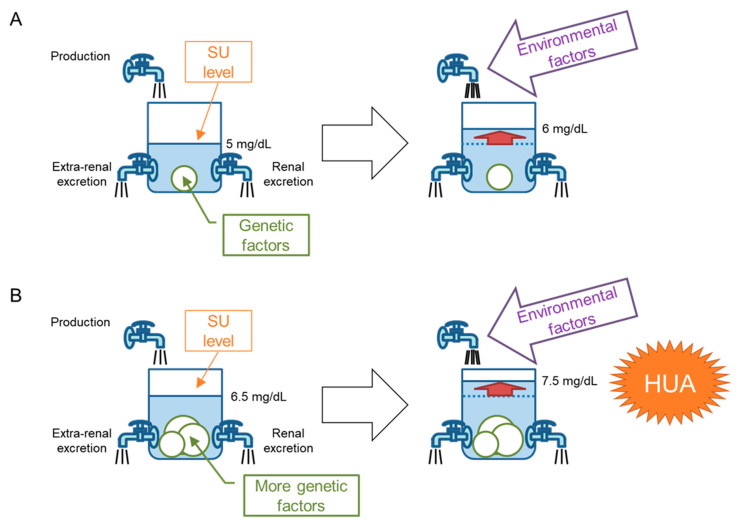
“Bucket-and-balls” theory. Genetic factors from *ABCG2* variants reportedly had a stronger effect on the progression of hyperuricemia in the Japanese population than common environmental factors. We propose here a “bucket-and-balls theory” to explain this apparent contradiction in the progression of hyperuricemia. The serum urate (SU) level in the human urate pool is regulated by the balance among uric acid production, extra-renal, and renal urate excretion. This can be compared to the water level (SU level) in a bucket (urate pool) and faucets (uric acid production and excretion). Genetic factors due to urate transporter genes that favor hyperuricemia correspond to changing faucet sizes, typically becoming smaller, which is equivalent to putting balls in a bucket, resulting in an elevated water (SU) level ((**A**), left). The water (SU) level will also be raised by environmental factors such as the overproduction of uric acid due to a purine-rich diet ((**A**), right). Since Japanese and indigenous Asia-Pacific populations carry strong genetic factors, equivalent to having more balls in the bucket, their SU levels will be higher than those with fewer genetic factors ((**B**), left); relatively small environmental factors are therefore enough to elevate the SU level to >7 mg/dL (>420 μmol/L), that is, to cause hyperuricemia (HUA; (**B**), right). This theory is useful not only for explaining why genetic factors show a stronger effect size than environmental factors but also for patient education on gout and hyperuricemia, which stresses the importance of controlling environmental factors.

Nakayama et al. [64] demonstrated that genetic factors from dysfunctional variants of *ABCG2* had a stronger effect on hyperuricemia progression in the Japanese population than did typical environmental factors. In their study [64], the population attributable fraction (PAF, also known as population attributable risk percent; PAR%) of ABCG2 dysfunction, which indicates the percentage of hyperuricemic patients originating from ABCG2 dysfunction in the population, was calculated to be 29.2%. This was much higher than overweight/obesity (BMI ≥ 25.0; PAF = 18.7%), heavy drinking (>196 g/week (male) or >98 g/week (female) of pure alcohol; PAF = 15.4%), and aging (≥60 years old; PAF = 5.74%). It appears counterintuitive that the genetic contributors to a lifestyle-related disease have greater effects than environmental factors. Here, we propose a “bucket-and-balls theory” to explain this apparent contradiction for hyperuricemia progression (Figure 4). The SU level in the human urate pool is regulated by the balance of uric acid production, extra-renal excretion, and renal excretion. This can be compared to the water level (SU level) in a bucket (urate pool) and faucets (uric acid production and excretion), as shown in Figure 4. Genetic factors, including urate transporter genes that favor hyperuricemia, correspond to changing faucet sizes—typically to smaller ones—which is equivalent to putting balls in a bucket and results in an elevated water (SU) level (Figure 4A, left). The water (SU) level will also be raised by environmental factors such as uric acid overproduction due to a purine-rich diet (Figure 4A, right). Since certain individuals, such as Japanese and indigenous Pacific populations [16], have strong genetic factors or have more balls in the bucket, their SU level will be higher than those with fewer genetic factors (Figure 4B, left); relatively small environmental factors are therefore enough to elevate the SU level to >7 mg/dL (>420 μmol/L), that is, to cause hyperuricemia (Figure 4B, right). This theory is useful not only for explaining the results of Nakayama et al. [64] but also for gout and hyperuricemia patient education to stress the importance of controlling environmental factors.

## 6. Genetic Factors That Favor Dysuricemia in the Japanese Population

**Figure 5:**
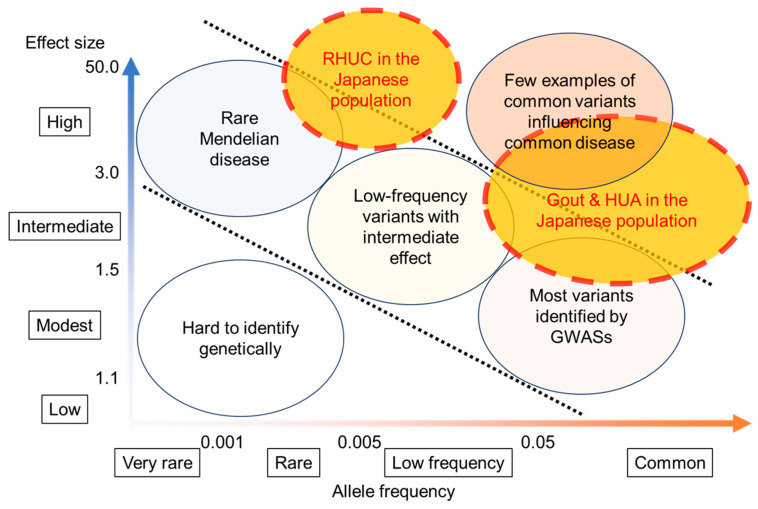
Strong and frequent genetic factors that favor dysuricemia in the Japanese population. As the allele frequency increases, its effect size generally decreases (adopted from [74,76]). Genetic studies of gout and hyperuricemia (HUA) in the Japanese population have revealed a “common disease-common variant” model with a higher effect size than other common diseases. Furthermore, the founder effect in the Japanese population causes relatively frequent hereditary renal hypouricemia (RHUC) with a higher effect size. Taken together, strong and frequent genetic effects on serum urate levels result in unique distributions of dysuricemia (gout/HUA and RHUC) in the Japanese population.

Recent comprehensive genetic analyses such as genome-wide association studies (GWASs) have identified many gout- and SU-associated loci. Multiple novel and significant loci were revealed by GWASs of clinically defined gout patients [17,65,66,67,68,69] and by GWASs of SU in ordinary populations [70,71,72,73]. As expected, many transporter genes such as *URAT1*/*SLC22A12*, *GLUT9*/*SLC2A9*, *ABCG2*/*BCRP*, *NPT1*/*SLC17A1,* and *OAT10*/*SLC22A13* were identified as having an association with gout and SU in addition to several enzyme genes, including the *ALDH2* gene, which has a pivotal role in alcohol metabolism. It is a distinguishing characteristic of gout-associated loci that their odds ratios (ORs) sometimes exceed 2.0, much higher than for loci of other common diseases, such as diabetes mellitus and hypertension, whose ORs range from 1.1 to 1.5 [74]. Single-nucleotide polymorphism (SNP)-based heritability of gout types was estimated to reach 35.5% [75]. With these facts taken together, Japanese populations have unique and remarkable characteristics with respect to dysuricemia (Figure 5). Figure 5 displays the general relationship between the allele frequency of disease-associated loci and their effect size [74,76]; the more common the allele frequency, the less its effect size increases. GWASs of gout and SU in the Japanese population, however, have revealed the “common disease-common variant” model to have a much higher effect size than other common diseases. Furthermore, the founder effect in the Japanese population causes relatively frequent RHUC, affecting approximately 0.3% of its population [26]. RHUC is, therefore, a more frequent Mendelian disease with a high effect size in Japanese, although it is a rare Mendelian disorder worldwide. The Japanese population thus has an ideal genetic background for investigating the pathophysiology of dysuricemia, as well as the physiology of uric acid and its handling.

## 7. Secondary Dysuricemia

In addition to primary hyperuricemia and gout, as described above, patients with secondary hyperuricemia/gout caused by pre-existing conditions (Table 1) [63] could sometimes be observed. Increased cell cycle and proliferation by malignant tumors, as well as decreased urate excretion by kidney dysfunction, could cause persistent secondary hyperuricemia in addition to transient hyperuricemia caused by a purine-rich diet, alcohol consumption, heavy exercise, and dehydration. Drugs such as diuretics (thiazide and furosemide), antiphthisics (pyrazinamide and ethambutol hydrochloride), cyclosporin, and theophylline can also induce secondary hyperuricemia. Tumor lysis syndrome by anticancer drugs could be regarded as another type of drug-induced hyperuricemia. As rare Mendelian disorders, Lesch–Nyhan syndrome (LNS; MIM 300322), a type of autosomal dominant tubulointerstitial kidney disease (ADTKD1; MIM 162000: previously called familial juvenile hyperuricemic nephropathy: FJHN), and glycogen storage disease type VII (GSD7; MIM 232800) are examples of hereditary secondary hyperuricemia and gout.

Except for RHUC and xanthinuria, almost all hypouricemia tends to be observed as secondary hypouricemia that is caused subsequent to other pathological conditions, diseases, and/or syndromes. As shown in Table 1 [8], the differential diagnosis should be considered as one of the syndromes that include inappropriate secretion of antidiuretic hormone (SIADH), Fanconi syndrome, and uricosuric agents in addition to starvation or emaciation. Malignant tumors and diabetes could also cause hypouricemia due to increased renal urate excretion. Uricosuric agents such as allopurinol, benzbromarone, and probenecid can induce hypouricemia [8,77]. Other rare Mendelian diseases, such as Wilson’s disease (WND; MIM 277900) and purine nucleoside phosphorylase (PNP) deficiency (MIM 613179), could cause hypouricemia.

## 8. Relationship between Diseases and Dysuricemia

SU levels have been epidemiologically proven to be related to several common diseases. Figure 1 is the summary of three patterns of such relationships between SU levels and other diseases: gout pattern, ND pattern, and CKD and CVD pattern.

### 8.1. Gout Pattern: Crystal Formation and Pro-Oxidative Effects

Diseases showing gout patterns (Figure 1) cause the formation and deposition of urate crystals. Because gout is caused by MSU crystal formation after prolonged hyperuricemia, the relationship between SU and gout is clearly high in the hyperuricemic area (SU > 7 mg/dL, or 420 μmol/L) [5], which results from the low solubility (6.8 mg/dL, or 400 μmol/L) of uric acid in human serum [9]. In other words, the gout pattern will depend upon the physical state of uric acid. This pattern appears to be the basis of the idea of “the lower, the better”, but it is incorrect because there are two more patterns as described below.

### 8.2. ND Pattern: Anti-Oxidative Effects

In contrast to the gout pattern, the ND pattern (Figure 1) indicates a relationship of “the higher, the better”, likely due to the anti-oxidative effect of uric acid. NDs such as Alzheimer’s disease (AD) and Parkinson’s disease (PD) are common diseases that can result in dementia. Two nationwide studies, from the U.K. and Taiwan, reported a lower incidence of AD and non-vascular dementia, respectively, in patients with and without gout [78,79,80]. ABCG2 dysfunction indeed hastened gout onset but was also significantly associated with later PD onset [81]. This report proposed a model in which ABCG2 dysfunction in the blood–brain barrier (BBB) plays an important neuroprotective role against increased urate levels in the central nervous system (CNS), together with higher SU levels due to ABCG2 dysfunction in the kidney and intestine [81]. In addition to a cohort study with over 18,000 males, which showed that high SU decreased PD risk [82], meta-analyses report low SU to increase the risk of AD and PD with dementia but not vascular dementia [83]. Another meta-analysis revealed a significant relationship with a large effect size for lower SU in amyotrophic lateral sclerosis (ALS) patients [84]. A prospective controlled inception cohort study indicated that gout patients had a lower risk of death due to dementia [85]. Meta-analyses of cerebral stroke also showed the ND pattern [86,87]. Based on these and other data, the European League Against Rheumatism (EULAR) recommended, in its 2016 gout management guideline, not to lower the SU level to <3 mg/dL (<180 μmol/L) continuously for several years [88].

While these epidemiological reports prove the ND pattern to have neuroprotective effects, not all studies reach this conclusion. For example, three Mendelian randomization (MR) studies, which modeled randomized controlled tests (RCTs) using a genetic approach, did not demonstrate any causality between SU or gout on the susceptibility of PD [89,90] or AD [91], respectively. An RCT of the effect of urate-elevating inosine on early PD progression did not result in a significant difference in the rate of clinical disease progression [92]. Further research is necessary to elucidate the effects of uric acid on NDs.

### 8.3. CKD and CVD Pattern: Combination with Gout and ND Patterns

The “CKD & CVD pattern” generates a J-curve or a U-curve (Figure 1), which indicates “the more normal, the better”. This pattern results from a combination of gout and ND patterns, that is, a balance between crystal formation and the pro- and anti-oxidative effects described above. Vascular-focused common diseases such as CKD and CVD tend to follow this pattern, probably because the XOR function as described below has effects on and around the vascular endothelium: (1) XOR produces ROSs as a pro-oxidant (Figure 2); (2) uric acid reduces ROSs, acting as an anti-oxidant (Figure 2); (3) the nitrite reductase activity of XOR generates nitric oxide (NO), which contributes to vasodilation and regulation of blood pressure [7]; (4) the NADH oxidase activity of XOR produces ROSs [7]; and (5) urate crystals can be deposited in blood vessels, possibly acting as a component of atherosclerotic plaque [93]. There are, however, some inconsistent reports on the harmful effects on the vascular system via XO, which produces ROSs. For example, Kusano et al. [94] produced XDH-stable and XO-locked knock-in mice and found no noticeable difference in survival rates between XOR mutant mice and wild-type mice. Further studies will be needed on this point.

Nevertheless, Nardi et al. [93] also revealed the increasing risk of cerebrovascular events with rising SU levels, suggesting that the deposition of urate crystals in carotid plaques could participate in the mechanism of stroke. Indeed, Konta et al. [95] reported all- and CVD-caused mortality to show a J-shaped association with SU level using a Japanese nationwide database of 500,511 subjects, followed up for seven years. J-curve results were also obtained from the PIUMA Study of 1720 CVD patients with hypertension [96] and from a nationwide community-based cohort study of 2081 nonfatal strokes in 155,322 subjects [97]. Nakayama et al. [98] identified both high and low SU levels as risk factors for CKD incidence in a sample of 138,511 middle-aged men and women. Kuwabara et al. also showed hypouricemia to be associated with a history of kidney disease in males, using 90,143 Japanese outpatients [99].

The CKD and CVD pattern might be predictive of mortality. With a South Korean cohort of 375,163, Cho et al. found a U-shaped association between all-, CVD-, and cancer mortality [100]. In addition to these mortalities, Hu et al. also revealed a similar association in respiratory-disease mortality from 9118 U.S. adults in the National Health and Nutrition Examination Survey (NHANES) dataset [101]. Hyperuricemia is reportedly a risk factor for cardiovascular and all-cause mortality in addition to kidney dysfunction in patients at cardiovascular risk [102], suggesting that CKD, together with hyperuricemia, might raise the risk of CVD progression.

It is of interest that a U-shape was also generated for COVID-19 severity, i.e., the risks inherent in invasive mechanical ventilation, in 1523 Japanese patients [103]. However, MR studies revealed the causality of SU and gout on COVID-19 in the Japanese population but not in Europeans, suggesting a difference in genetic backgrounds between these ancestries [104]. Further analyses will be necessary.

### 8.4. Range of Normouricemia as Optimal SU Level

Considering these patterns above, how should we range normouricemia or “optimal SU”? From the viewpoint of “gout pattern”, the definition of hyperuricemia is set at SU of >7.0 and >6.8 mg/dL (>420 and >400 μmol/L), irrespective of gender in the Japanese [63] and American College of Rheumatology (ACR) [105] guidelines, respectively, due to the low solubility (6.8 mg/dL, or 400 μmol/L) of uric acid in human serum [9]. From the “ND pattern”, maintaining SU at ≥3 mg/dL (≥180 μmol/L) would be better, as EULAR recommends [88]. Research on the “CKD & CVD pattern” [95,96,97,98,100,101] indicates that the SU range at the lowest disease risks depended on sex and was estimated to be approximately 4–7 mg/dL (240–420 μmol/L) for males and 3–6 mg/dL (180–360 μmol/L) for females, although set SU ranges differed in each study and were impossible to integrate. Furthermore, the RHUC guidelines strongly recommend that individuals with an SU of ≤2.0 mg/dL (120 μmol/L) should be considered to have RHUC from epidemiological evidence [8], and an additional genetic and epidemiological report [26] suggests that males and females with SU of ≤3.0 and ≤2.0 mg/dL (≤180 μmol/L and ≤ 120 μmol/L), respectively, should be considered to have RHUC.

Taken together, we propose that 4 < SU ≤ 7 mg/dL (240 < SU ≤ 420 μmol/L) for males and 3 < SU ≤ 6 mg/dL (180 < SU ≤ 360 μmol/L) for females should be regarded as normouricemia, or optimal SU, to take best advantage of both the roles played by uric acid (Figure 1).

## 9. Conclusions with Future Research/Clinical Questions on Dysuricemia

In this review, the dual roles of uric acid, that is, an anti-oxidative protective effect and a pro-oxidative and/or a harmful crystal-forming effect, are described to support our proposition of this novel disease category, “dysuricemia”. However, there are still many research and clinical questions on dysuricemia to be answered in future research. Below are example questions.

➢ What causes EIAKI in RHUC patients? One hypothetical mechanism of EIAKI suggests that lowered anti-oxidative effect in RHUC patients causes renal vasopressin by exercise-induced ROSs from XOR; based on this hypothesis, some case studies report the effectiveness of allopurinol or febuxostat (XOR inhibitors) administration in preventing EIAKI [106,107,108,109]. However, convincing evidence for their efficacy is lacking [8];
➢ While some epidemiological studies on NDs support the “ND pattern”, several other studies (described above) do not. Research into the effects of low SU against NDs should be conducted to elucidate the effects of urate on NDs;
➢ Which comes first, dysuricemia, CKD, or CVD? It is also possible that SU is simply a marker of these diseases. Further investigation of their causality by SU should be determined from the viewpoint of the anti-oxidative, pro-oxidative, and crystal-forming effects of urate;
➢ Why are females more vulnerable to SU? It is known that female hormones decrease SU levels [5]. Our previous studies do, in fact, reveal sex differences in SU in the order of 1–1.5 mg/dL (60–90 μmol/L) [26,54]. The optimal SU range differs between the sexes by 1 mg/dL (60 μmol/L), so females can be concluded to be more vulnerable to SU at the same SU level as males.

Dysuricemia, like dyslipidemia and diabetes mellitus, is a disorder of energy homeostasis. Here, we again propose the novel disease concept of “dysuricemia” to prevent pathogenesis from excessively high or low SU and suggest that its spectrum be interpreted as a single disease category to be able to take optimal advantage of the dual nature of uric acid.

## Figures and Tables

**Table 1:** Diseases and factors that result in dysuricemia.

	Uric Acid Production	Urate Excretion	Production and Excretion
Hyperuricemia	Renal overload type (overproduction type and extra-renal underexcretion type *)	Renal underexcretion type	Combined type
	➢ Purine rich diet ➢ Dysfunctional ABCG2 * ➢ Lesch-Nyhan syndrome/Kelley-Seegmiller syndrome ➢ Phosphoribosylpyrophosphate synthetase (PRS) superactivity ➢ Glycogen storage disease type VII (also known as muscle phosphofructokinase (PFK) deficiency, myogenic hyperuricemia, and Tarui Disease) ➢ Malignant neoplastic disease ➢ Non-neoplastic disease (e.g., psoriasis) ➢ Tumor lysis syndrome ➢ Rhabdomyolysis ➢ Hypothyroidism ➢ Drugs (e.g., theophylline)	➢ Dehydration ➢ Kidney disease (e.g., chronic kidney disease, polycystic kidney) ➢ Autosomal dominant tubulointerstitial kidney disease caused by mutation in UMOD or REN [ADTKD-*UMOD* or ADTKD-*REN*; formerly called familial juvenile hyperuricemic nephropathy (FJHN) or familial juvenile hyperuricemic nephropathy type 2 (FJHN2), respectively] ➢ Hyperlactacidemia ➢ Drugs [e.g., diuretics (thiazide and furosemide), antiphthisics (pyrazinamide and ethambutol hydrochloride), cyclosporin]	➢ Obesity ➢ Alcohol consumption ➢ Heavy exercise ➢ Pregnancy-induced hypertension ➢ Severe traumatic injury, burn injury ➢ Glycogen storage disease type I ➢ Drugs [e.g., Vitamin B3 (niacin)]
Hypouricemia	Underproduction type	Overexcretion type	Combined type
	➢ Xanthinuria (type I, type II) ➢ Molybdenum cofactor deficiency ➢ Purine nucleoside phosphorylase deficiency (PNP deficiency) ➢ PRS hypoactivity ➢ Idiopathic urate underproduction-type hypouricemia ➢ Severe hepatic injury ➢ Drugs (such as allopurinol) ➢ Emaciation (malnutrition)	➢ Renal hypouricemia (RHUC type I, type II) ➢ Fanconi syndrome ➢ Wilson’s disease ➢ Syndrome of inappropriate secretion of antidiuretic hormone (SIADH) ➢ Malignant tumor ➢ Diabetes mellitus ➢ Drugs (such as benzbromarone and probenecid) ➢ Pregnancy ➢ Intractable diarrhea	➢ Combination of underproduction and overexcretion types

Adopted from [63] for hyperuricemia and from [8] for hypouricemia. * In ordinary urinary tests, urate overproduction type is undistinguishable from extra-renal underexcretion type caused by dysfunctional ABCG2.
